# Physical principles of fluid-mediated insect attachment - Shouldn’t insects slip?

**DOI:** 10.3762/bjnano.5.127

**Published:** 2014-07-28

**Authors:** Jan-Henning Dirks

**Affiliations:** 1 Department of New Materials and Biosystems, Max Planck Institute for Intelligent Systems, Stuttgart, Germany

**Keywords:** adhesion, friction, insect biomechanics, tribology

## Abstract

Insects use either hairy or smooth adhesive pads to safely adhere to various kinds of surfaces. Although the two types of adhesive pads are morphologically different, they both form contact with the substrate via a thin layer of adhesive fluid. To model adhesion and friction forces generated by insect footpads often a simple “wet adhesion” model is used, in which two flat undeformable substrates are separated by a continuous layer of fluid. This review summarizes the key physical and tribological principles that determine the adhesion and friction in such a model. Interestingly, such a simple wet-adhesion model falls short in explaining several features of insect adhesion. For example, it cannot predict the observed high static friction forces of the insects, which enable them to cling to vertical smooth substrates without sliding. When taking a closer look at the “classic” attachment model, one can see that it is based on several simplifications, such as rigid surfaces or continuous layers of Newtonian fluids. Recent experiments show that these assumptions are not valid in many cases of insect adhesion. Future tribological models for insect adhesion thus need to incorporate deformable adhesive pads, non-Newtonian properties of the adhesive fluid and/or partially “dry” or solid-like contact between the pad and the substrate.

## Review

### How do insects adhere to surfaces?

More than 80% of the animal species in the world are arthropods [1], and amongst them insects can be considered probably the evolutionarily most successful group. For hundreds of millions of years they are inhabiting almost every part of the world, and different species have developed adaptations to environments with a wide range of temperatures, humidities and substrates.

For a long time the ability of insects and other arthropods to effortlessly walk up and down all kinds of natural and artificial surfaces has fascinated scientists and the underlying mechanisms have been debated since the early days of light microscopy. From “gluten-filled sponges”, the interlocking of fine hairs, suction cups and adhesive secretions, many hypotheses about insect adhesion have been proposed over the last two centuries [2–7].

In recent years more elaborate microscopes and better analytical tools have become available and the interest of biologists and engineers in biological and biomimetic adhesives has increased [8–13]. Imaging techniques such as atomic force microscopy (AFM) and various different types of scanning electron microscopy (SEM) now allow biologists to analyse sensitive biological samples on a nanometre scale. Nevertheless, the detailed mechanisms allowing insects to safely adhere to surfaces are still not fully understood.

In a previous paper we have discussed the general principles of insect adhesion and highlighted recent advances and open questions [14]. This review will focus on summarizing the key physical principles that are thought to determine the attachment forces. We will discuss in which aspects a commonly used simple “wet adhesion” model is sufficient and, in particular, in which it falls short in explaining the forces generated by the adhesive pads of insects.

### Hairy and smooth adhesive organs of insects

**Figure 1:**
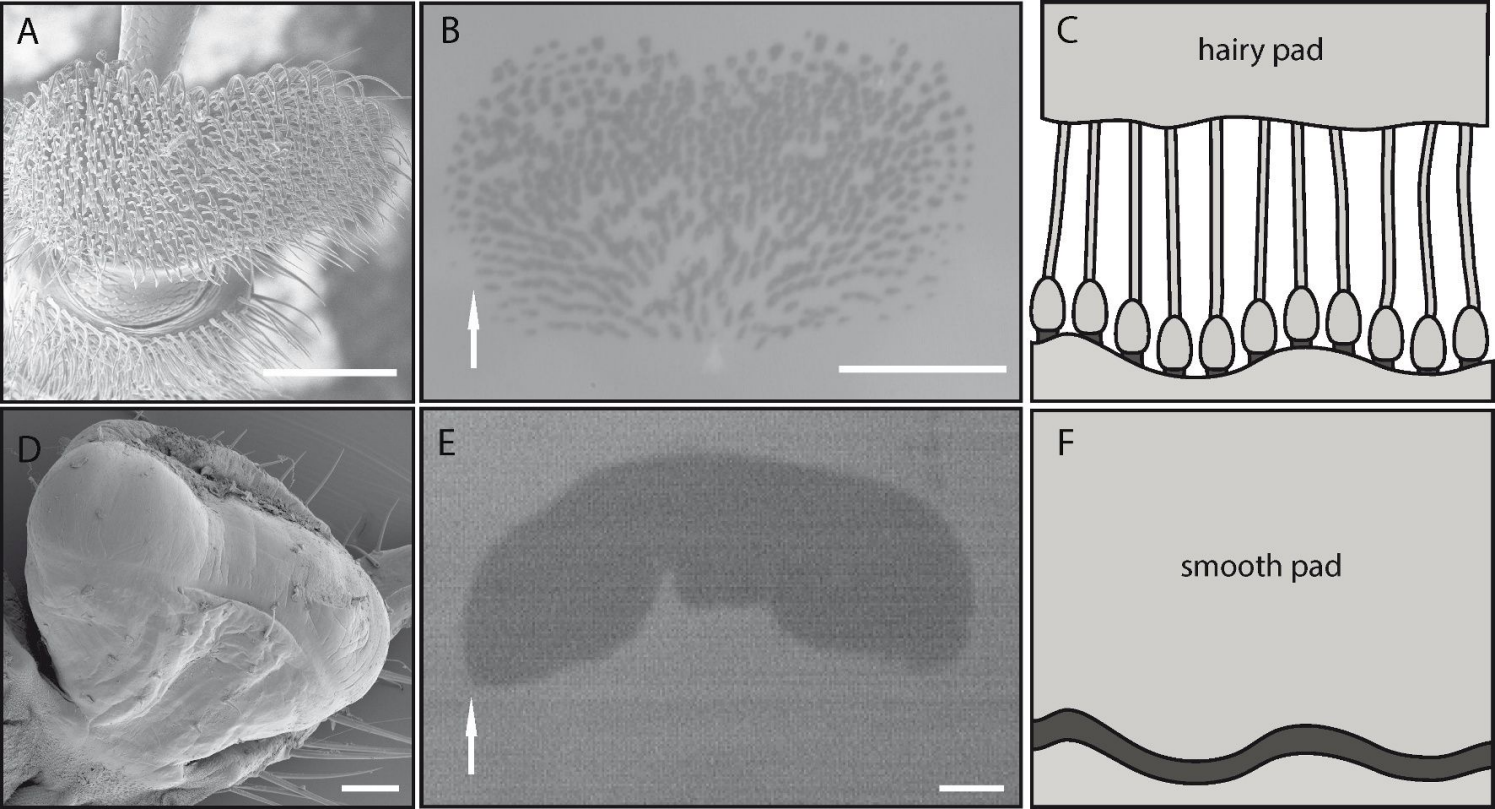
Adhesive pad morphology of a male dock beetle (*Gastrophysa viridula*, A) and an Indian stick insect (*Carausius morosus*, D). Epi-illumination can be used to visualise the contact area of the adhesive pad in contact with glass (B, E). Arrows indicate distal direction. Subfigures C and F show a schematic view of the close contact of the hairy and smooth pads to a rough substrate. Note that in both cases the contact zone is mediated by an adhesive fluid (dark). Images A, B, D and E adapted with kind permission of [16]. Copyright (2008) The Company of Biologists Ltd. All scale bars represent 100 μm.

One of the most basic biological micro-scale structures for mechanical interlocking to a substrate seems to be a claw. However, the potential use of claws is limited to compliant surfaces in which the claws can insert, or rough surfaces with asperities larger than the diameter of the claw tip [15]. Hence, to stick to smooth and stiff natural substrates, such as stones or leaves, insects and other arthropods have to use adhesive pads (Figure 1).

In general, the hairy adhesive pads on the feet of flies, beetles and spiders are densely covered with dense arrays of flexible setae (see Figure 1A) [16–22]. Although the setae of some beetles branch towards the tip, they only end in a single terminal element in most insects. These terminal elements can vary in shape and size, even within one tarsus or between the sexes of one species [23]. Recently it has been shown that in beetles the setae show a decreasing stiffness of the cuticle towards the tip of the setae [24]. Similar “hairy” structures can be found in many other biological adhesive pads such as lizards and spiders [25], indicating a general “favourable” design [8].

Despite the large number of species, only two types of adhesive systems have evolved in insects and other arthropods: “hairy” (fibrillar) and “smooth” pads [26]. Both systems provide attachment to rough and smooth surfaces by maximizing the contact area and achieving close contact [27–28]. In contrast to the hairy adhesives, smooth pads of insects increase the contact area by adapting as a whole to the surface roughness (Figure 1F, [29–32]). Smooth adhesive organs can be found in many insect groups such as ants, bees, stick insects, grasshoppers, true bugs and cockroaches. The larger number of insect groups with smooth pads, compared to the smaller number of groups with hairy pads, led to the suggestion that smooth pads probably appeared earlier in insect evolution and represent a more “basic” evolutionary adhesive structure [22]. In addition, the structural diversity of the smooth pads in different insect orders has lead to the suggestion that smooth adhesive pads have independently evolved several times [27,33]. Recently it has been shown that insects with smooth adhesive pads can also possess hairy “friction pads” with special morphological adaptations [34].

Despite their different morphology, hairy and smooth adhesive pads of insects have in common that they both secrete an adhesive fluid into the contact zone [14,16]. For the smooth pads of stick-insects, cockroaches and ants it has been shown that this adhesive fluid is a two-phasic microemulsion consisting of a hydrophilic, volatile dispersive phase within a hydrophobic, persistent continuous phase [30,35]. In hairy pads, with notably smaller contact points of each seta (and thus an even more complicated analysis of the foot secretion [36]), the detailed chemical composition is still a subject of investigation. Although it has been shown that the composition of the lipophilic phase of the secretion is similar to the composition of the cuticular hydrocarbon layer [37].

To understand the physical principles that enable the attachment of such fluid-mediated insect foot pads onto various substrates, one has to start with a simple model. In general, the fluid-mediated smooth and hairy adhesive organs of insects have to generate both adhesion forces perpendicular to the substrate and friction forces parallel to the substrate. The tribological models for both “types” of attachment forces will be considered separately in the following.

### Fluid-mediated adhesion

**Figure 2:**
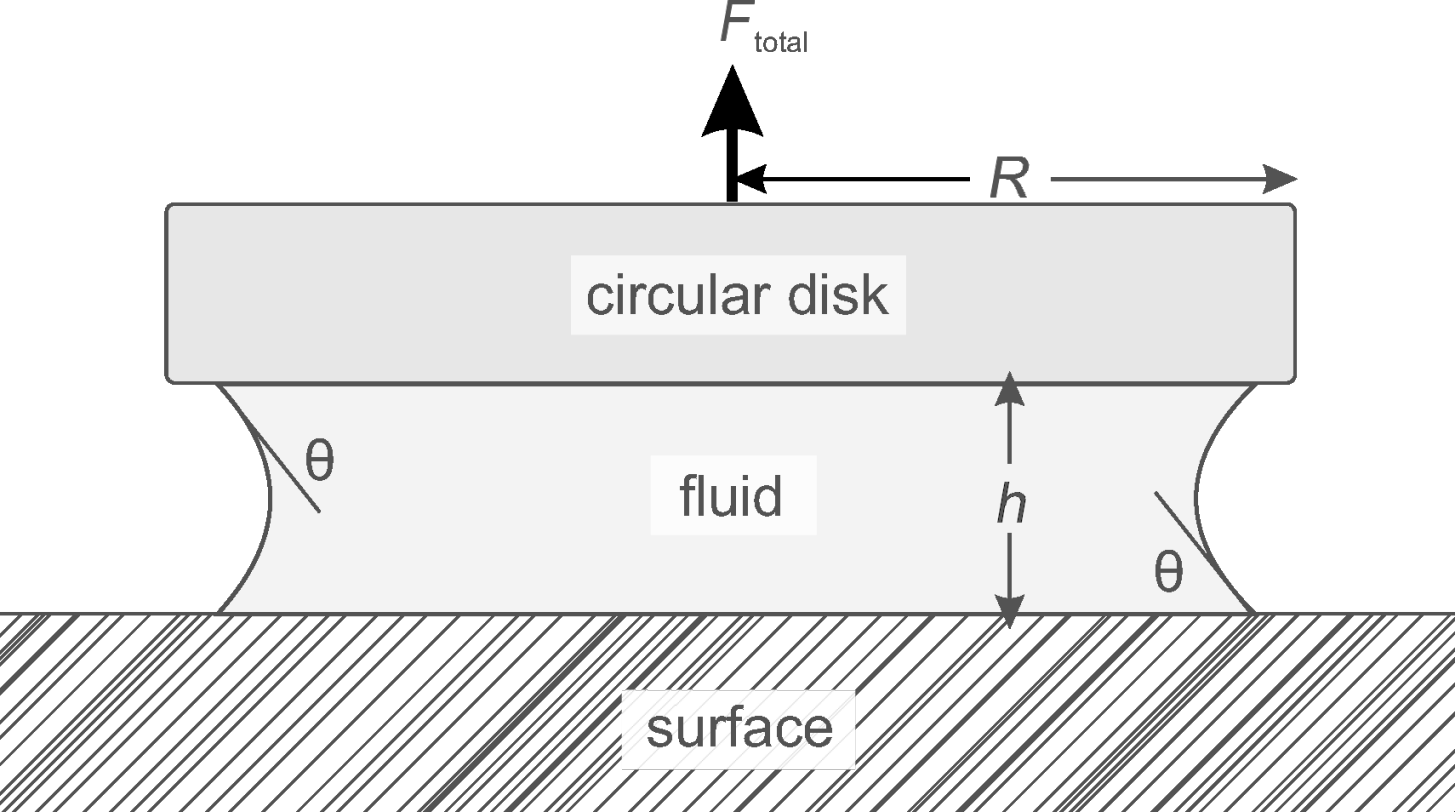
Schematic illustration of a simple fluid-mediated attachment model. The contact of a circular rigid disk (radius *R*) and a plane rigid surface is mediated by a thin fluid layer (height *h*). The contact angle of the fluid to the surface and the disk θ is assumed to be identical in this case.

For simplicity, the contact zones of both smooth and hairy adhesive pads are often modeled by using a “wet-adhesion model” consisting of a smooth, undeformable disk and substrate with a mediating continuous fluid-layer (see Figure 2 and [30,38–42]).

In this simple model, the total adhesive force is basically the sum of three components: the surface tension of the fluid, the Laplace pressure (both often combined as “capillary forces”) and the viscous forces, often called “Stefan adhesion” [43–44] (see below in Table 1).

[1]
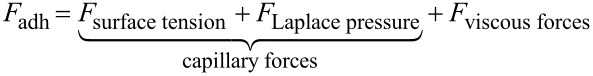


Based on a few assumptions and further simplifications (such as equal contact angles θ of the mediating fluid layer to the surface and the disk), the adhesive forces generated by such a “wet adhesion” model in Equation 1 can be estimated by using

[2]
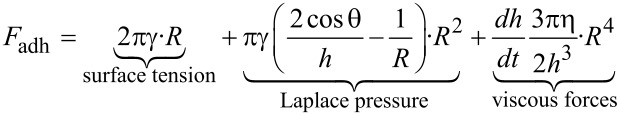


By using a few experimentally accessible parameters such as mediating fluid height, viscosity, and contact angle (for a more detailed review on the challenges on measuring these parameters see [14]), this model can then be used to discuss and qualitatively predict a few characteristic “features” of insect adhesion (see Table 1). For example, looking only at the Laplace term in Equation 2

[3]
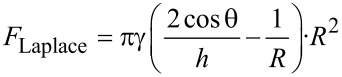


one can predict that an increasing fluid height *h* between adhesive pad and surface would result in decreasing capillary adhesive forces. As a consequence, insects and all organisms with fluid-mediated attachment organs should minimize the secretion of adhesive fluid into the contact area to increase capillary adhesion on smooth surfaces.

However, and this is where the simple “wet adhesion model” starts to fall short when used to model insect attachment, only very few natural surfaces are actually smooth. Experimental studies indeed show that the adhesive fluid actually plays a more important role in increasing adhesion on rough surfaces by filling gaps between the pad and the surface, thereby maximizing contact area and adhesion to rough substrates [45–46]. This has been shown by studies in which adhesive fluid was either experimentally accumulated or depleted. In smooth and hairy adhesive systems an additional fluid volume decreased friction and adhesive forces on smooth surfaces, as predicted by the simple model. When forces were measured on rough surfaces however, accumulating adhesive fluid increased adhesion, as the additional fluid compensated the surface roughness and thus increased the effective contact area [16,46]. In addition, the presence of adhesive fluid has been shown to improve the self-cleaning of the adhesive pads in comparison to dry adhesive pads [47–48].

Besides the “smooth vs rough” limitation, the simple model also bears several additional notable problems, in particular in light of the stiffness and deformability of the adhesive pads. For example, the capillary term in Equation 2 is only valid in the case of rigid, stiff surfaces in contact. In the case of insect (and tree frog) attachment, with very smooth and adaptable pads [49–50], it is very questionable whether this assumption is justified. In fact, recent and more comprehensive tribological models show that for certain ratios of adhesive pad size and stiffness, the Young’s modulus of the adhesive pad can play an important role in determining the capillary adhesion [51–52]. In these models the overall capillary force is taken as the sum of the capillary attraction and the counter-acting elastic repulsion of the deformed pad/substrate (which depends on the elastic modulus). In simple terms, a softer adhesive pad/substrate (with a lower elasticity) deforms more easily at a given external force, resulting in a larger contact area. This larger contact area again increases the radius of the mediating meniscus of the liquid (the liquid is pressed towards the outside of the pad), which then increases the capillary force. This extended capillary model might add an explanation why some insects (and tree frogs) have soft toe pads [51].

It should also be noted that in particular in the context of insect adhesion the mechanics described by the third viscous forces, or “Stefan adhesion” term, are a rather substantial simplification of the processes determining the viscous adhesion between the adhesive pad and the substrate. The mechanism proposed by Stefan also assumes undeformable surfaces and uniform centripetal flow of the mediating fluid [53]. Both assumptions are very likely to be violated in the case of the very compliant smooth insect adhesive pads on micro-rough substrates [26,54]. Hence, in this context the term “Stefan adhesion” should only be used with great care. Also, it is important to note that according to the model, the forces generated by the viscous properties of the mediating fluid volume should decline over time. In a static adhesive pad with a Newtonian fluid, only the (negligible) surface tension and the small Laplace pressure would thus determine the overall adhesive force of the insect foot (Table 1).

**Table 1:** Summary of physical principles often used to model the attachment forces generated by the adhesive feet of insects. However, based on this simple model (besides other limitations discussed in detail in the text), insects should not be able to generate sufficient static friction forces to prevent slipping.

	fluid-based attachment				

	adhesion			friction	
	
factor	“surface tension”	“Laplace pressure”	viscous force	“surface tension”	hydrodynamic lubrication

contact area	×	×	×	×	×
fluid height	—	×	×	—	×
contact angle	×	×	—	—	—
surface tension	×	×	—	×	—
Viscosity	—	—	×	—	×
dynamics	—	—	change of fluid height	—	sliding velocity

### Fluid-mediated friction forces

In a fluid mediated system with a continuous Newtonian fluid film, the friction forces between the substrates can in general be described by using two basic principles: the surface tension of the mediating fluid and the laws of hydrodynamic lubrication [55–56].

#### Surface tension

The contribution of the surface tension of the mediating fluid to friction forces can be estimated by using a simple model of a mercury thread moving through a glass tube [55]. For a simplified model with a circular contact area with radius *r*, and α_1_ and α_2_ as the leading and trailing edge contact angles [57], the retentive “friction” force *F* acting on the mercury can be described by using

[4]
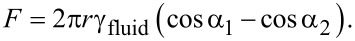


However, the contribution of surface tension towards the generation of friction forces is very limited. Looking at the adhesive fluid secreted by the Indian stick insect *Carausius morosus* with an estimated surface tension of 27 mNm^−1^ [58] and a contact area of 0.1 mm^2^ [16], the maximal possible shear stress before sliding occurs (with cos α_1_ − cos α_2_ ≤ 2) would be only approx. 0.38 kPa. This value (and the corresponding value for smaller contact areas) is several orders of magnitude smaller than the shear stresses measured in smooth adhesive pads of the stick insects, cockroaches and ants [16,46,57,59]. Surface tension alone is thus unlikely to explain the high friction forces generated by the adhesive pads of insects.

#### Hydrodynamic or boundary lubrication?

Similar to the viscous forces in adhesion, the “hydrodynamic lubrication” friction model takes into account the viscosity of the mediating fluid layer. Two parallel smooth surfaces with a distance of *h* sliding at a velocity *v* relative to each other generate the friction force

[5]
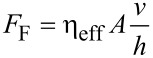


where *η*_eff_ is the effective viscosity of the mediating fluid layer and *A* the size of the contact area. Again, similar to the time-dependent viscous adhesion (Equation 2), the *v*/*h*-term in Equation 5 shows that a simple fluid mediated system at rest should not be able to generate any static friction. Based on viscosity estimations from dewetting processes (40 to 150 mPa), Federle et al. showed that the hydrodynamic friction forces generated by a continuous fluid film of 90 nm height would be one order of magnitude smaller than the shear stresses observed in adhesive organs [30]. Thus it is not yet clear how insects with a fluid-mediated adhesive pad generate the observed friction forces preventing them from sliding down smooth vertical substrates. As a quick look at the nearest window pane can confirm, insects can actually safely adhere to smooth vertical substrates. This indicates that a simple “continuous” and Newtonian fluid-layer model is not a valid model for the friction forces of insect pads [57]. Indeed, experimental results suggest that thixotropic (shear-thinning) non-Newtonian properties of the secretion could explain the presence of high static friction forces [46,60].

**Figure 3:**
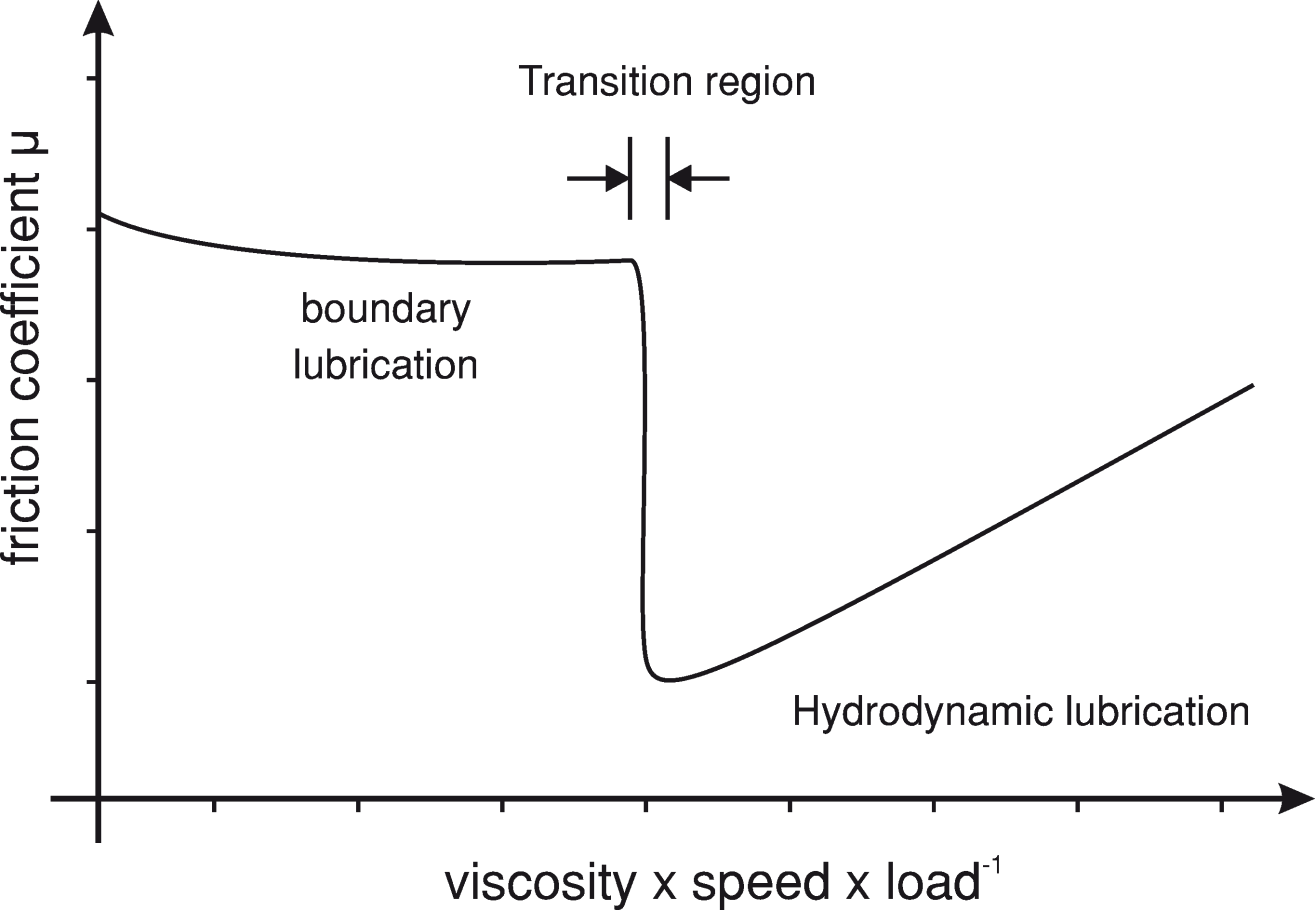
Regions of hydrodynamic and boundary lubrication of two fluid mediated smooth surfaces (Stribeck curve). With decreasing sliding velocity and increasing load the mediating fluid film becomes thinner, allowing regions of direct contact between the two surfaces. Within the transition region the friction coefficient rapidly increases, usually to a value smaller than unlubricated surfaces. This region is also referred to as elasto-hydrodynamic lubrication. It is not yet fully understood in which of the two “regions” the adhesive organs of insects operate.

A second, yet to be tested, hypothesis to explain the observed high friction forces is the formation of local “dry” rubber like direct contacts between the adhesive pad and the substrate [61]. The classic hydrodynamic model only describes the friction observed with relatively “thick” layers of lubricant (≥0.5 μm, [62]), where neither the specific surface properties (roughness, surface energy) nor Amonton’s law are involved [63]. Friction forces generated by fluid layers thinner than 5 to 10 molecular layers are usually modeled by using the more complex boundary lubrication theory, in which a decreasing film height and increasing number and area of direct contacts between the two substrates actually increase the friction coefficient [56,64–65]. The range between the hydrodynamic model and the boundary lubrication model, in which the mediating fluid layer is still lubricating the contact, however allows a weak interaction between the surfaces, is mostly referred to as elasto-hydrodynamic lubrication (Figure 3 and [66–67]). Within this range, the mediating fluid film can become unstable and areas with “dry” solid-like contact can form as a result of local “dewetting” and increased friction forces. Indeed, a similar mechanism has been proposed for the fluid-mediated adhesive toes found in tree frogs, for which force measurements and interference reflection microscopy results indicate that boundary friction might be responsible for the friction [49,68]. However, although first results indicate that the mediating fluid layer in insect pads might be thinner than previous estimates [69], so far there is no experimental evidence for the occurrence of dewetting or direct contacts between adhesive pad and surface in smooth or hairy adhesive organs of insects [14,30–31,46].

## Conclusion

Even after many years of research, several of the fundamental physical properties enabling insects to safely adhere to surfaces are still not fully understood. Although simple fluid-mediated models based on capillary and viscosity are still often used to estimate the attachment forces generated by insect feet, newer experimental data and more comprehensive tribological models suggest that many aspects of these classic “microscopic” attachment models fall short in fully explaining the forces generated by the fluid-mediated adhesive pads of insects.

To fully understand the physical principles of insect adhesion, one thus has to extend (or even replace) the “classic” models of friction and adhesion to incorporate for example deformable pads or non-Newtonian properties of the adhesive fluid. In particular in respect to friction forces one has also take into account possible more complex nano-tribological models, with boundary lubrication or other mechanisms resulting in points of “dry” contact between the pad and the surface.

Further fundamental experimental work, in particular a more accurate in vivo measurement of the height of the mediating fluid and high-resolution single-leg force measurements on smooth substrates with well known physical properties are required to answer these questions [14].
